# Comparative analysis of the visual performance after implantation of the toric implantable collamer lens in stable keratoconus: a 4-year follow-up after sequential procedure (CXL+TICL implantation)

**DOI:** 10.1136/bmjophth-2017-000090

**Published:** 2017-09-28

**Authors:** Farideh Doroodgar, Feazollah Niazi, Azad Sanginabadi, Sana Niazi, Alireza Baradaran-Rafii, Cyrus Alinia, Eznollah Azargashb, Mohammad Ghoreishi

**Affiliations:** 1 Ophthalmology Department, Eye Research Center Tehran University of Medical Sciences, Tehran, Tehran, Iran; 2 Shahid Beheshti University of Medical Sciences and Health Services , Chamran Highway, Daneshjoo Street, Tehran, Iran, Thran, Tehran, Iran; 3 Shahid Beheshti University of Medical Sciences and Health Services , Chamran Highway, Daneshjoo Street, Tehran, Iran, Tehran, Tehran, Iran; 4 Shahid Beheshti University of Medical Sciences and Health Services , Chamran Highway, Daneshjoo Street, Tehran, Iran, Tehran, Tehran, Iran; 5 Department of Public Health, Urmia University of Medical Sciences, Urmia, West Azerbaijan, Iran; 6 Shahid Beheshti University of Medical Sciences and Health Services , Chamran Highway, Daneshjoo Street, Tehran, Iran, Tehran, Tehran, Iran; 7 Ophthalmology Department, Isfahan University of Medical Sciences, Isfahan, Isfahan, Iran

**Keywords:** keratoconus, cross-linking, toric implantable collamer lens (TICL)

## Abstract

**Aims:**

To report on 4-year postoperative visual performance with the toric implantable collamer lens (TICL) for stable keratoconus after sequential procedure (corneal collagen crosslinking plus TICL implantation).

**Methods:**

Forty eyes of 24 patients with stable keratoconus with myopia between 0.00 and −18.00 dioptres (D) and astigmatism between 1.25 and 8.00 D were evaluated in this prospective interventional study (https://clinicaltrials.gov/ct2/show/NCT02833649). We evaluated refraction, visual outcomes, astigmatic changes analysed by Alpins vector, contrast sensitivity, aberrometry, modulation transfer functions (MTFs), defocus curve, and operative and postoperative complications.

**Results:**

At 4-year follow-up, 45% had 20/20 vision or better and 100% had 20/40 or better uncorrected visual acuity (UCVA). Vector analysis of refractive astigmatism shows that the surgically induced astigmatism (SIA) (3.20±1.46 D) was not significantly different from the target induced astigmatism (TIA) (3.14±1.42 D) (p=0.620). At 4 years postoperatively, none of the eyes showed a decrease in UCVA, in contrast to 24 eyes in which UCVA was increased by ≥1 lines, with contrast sensitivity and improvement in total aberrations and MTF value at 5 per degree (*p=0.004) after TICL implantation. The cumulative 4-year corneal endothelial cell loss was ≤5%. No patients reported dissatisfaction. At the end of follow-up, the vault was 658±54.33m (range, 500–711) and the intraocular pressure was 11.7±2.08 mm Hg. Occurrences of glare and night-driving troubles diminished after TICL surgery.

**Conclusion:**

The results from this standardised clinical investigation support TICL implantation from clinical and optical viewpoints in patients with stable keratoconus.

**Trial registration number:**

NCT02833649, Pre-results

**Key messages**

**What is already known about this subject?**

Patients with keratoconus have numerous biases in the diagnosis (lack of univariate or multivariate indices with 100% sensitivity and specificity for clinical definitions of keratoconus, grading or staging (index specificity is affected by the corneal topographer) and progression (even for Belin-Ambrosio Enhanced Ectasia Display, the normal measurement noise is required to be known to apply these parameters). Thus far, there is no consistent or clear definition of keratoconus, which affects the scientific power of studies. Consequently, this heterogenic disease requires numerous thorough studies with long-term follow-up.

**What are the new findings?**

- Evaluations about visual quality indexes, such as aberrometry, imply that the sensory system readapted to diffraction-limited ocular optics once toric implantable collamer lens (TICL) implantation occurred in patients with keratoconus.
- Selection of maximum tolerable myopic spherical components for patients with longer axial length with relevance to probable myopic regression postoperatively, and considering other warning signs, can yield better refractive result postoperatively.
- TICL implantation as a second procedure after crosslinking, in addition to refractive correction, improved contrast sensitivity in patients with keratoconus.

**How might these results change the focus of research or clinical practice?**

The clinical outcomes of the current study demonstrate the safety, efficacy and predictability of TICL in stable keratoconus. In addition, the use of this surgical method has had a profound effect on the quality of life of these patients. With all of this in mind, we believe that the experience gained in this case is worth sharing with those of our colleagues.

## Introduction

Keratoconus is a cone-shaped protrusion of the cornea that is derived from the Greek words kerato (cornea) and konos (cone). It is a progressive, non-inflammatory and bilateral thinning of the centreof the cornea and is found to be the most widely seen type of corneal ectasia.^1 2^ However, the exact mechanism by which it manifests in terms of progression, genetic heterogeneity and phenotypic diversity is not known, thereby resulting in a series of diverse diagnostic and treatment methods.^2 3^ Essential to each treatment is timing and applying the appropriate intervention method to each patient.^4^ The implantation of a phakic intraocular lens into the posterior chamber, as demonstrated by clinical observations, can be a desirable alternative to visual defects resulting from refractive errors in the state of keratoconus.^5^ The STAAR Surgical Visian ICL is an intraocular implant manufactured from a propriety hydroxylethylmethacrylate/porcine collagen-based biocompatible polymer material. The Visian ICL contains an ultraviolet (UV) absorber made from a UV-absorbing material. The Visian ICL has a plate haptic configuration with a central convex/concave optical zone and fuses a forward vault to minimise contact of the Visian ICL with the anterior capsule of the crystalline lens. The Visian ICL has been designed to be set completely inside the back chamber straight behind the iris and in front of the anterior capsule of the human crystalline lens, and when accurately situated, the lens works as a refractive component to progress vision.^6^

The toric implantable collamer lens (TICL) is used in adults aged 21–40 years for correcting myopia (−3.0 to −23.0 D) and astigmatism (≤6 D) in refraction, with stable refraction and anterior chamber depth (ACD) ≥3 mm. It is shown to be highly effective in preserving and improving best-corrected vision and preoperative values such as safety and stability. Nevertheless, issues that are debatable include the effects of TICL and the readaptation of the sensory system to diffraction-limited ocular optics after TICL implantation in patients with keratoconus.^5^ A comprehensive literature review of PubMed and Web of Science revealed that this investigation is the first one to study the long-term effectiveness of TICL on aberrations and contrast sensitivity in Iranian patients with stable keratoconus in January 2011.

## Methods

### Study design and subjects

**Figure 1:**
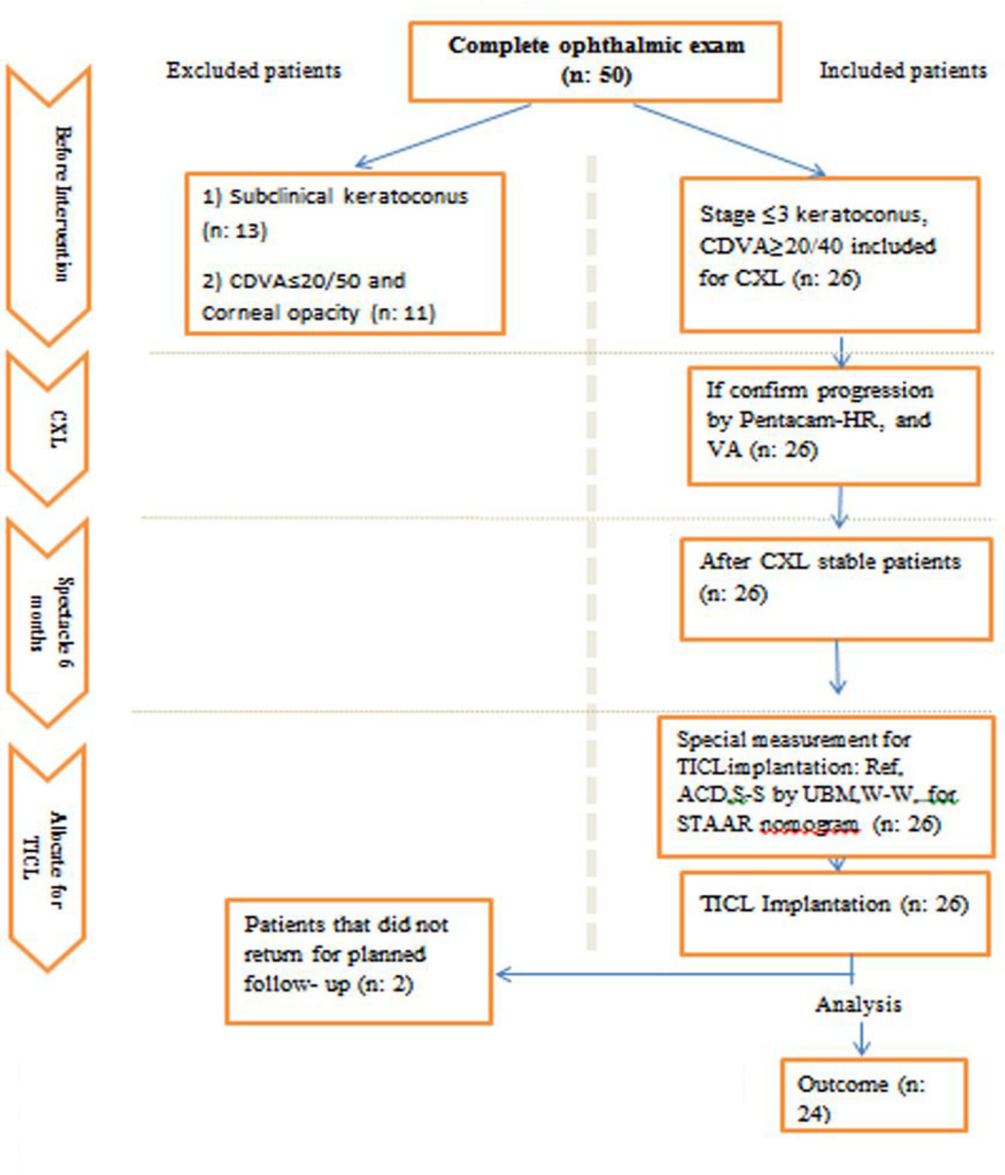
Eligibility assessment procedure. CDVA, corrected distance visual acuity; CXL, corneal collagen crosslinking; TICL, toric implantable collamer lens.

Twenty-four patients (40 eyes) age 25–38 years with normal systemic history and no physical signs of ocular disease other than keratoconic eyes were recruited in this study and treated with a sequential corneal collagen crosslinking (CXL) implantable collamer lens (ICL) (with at least a 12-month interval) procedure at the Negah Eye Hospital in Tehran, Iran (figure 1. These cases were selected using a non-random consecutive sampling method. The tenets of the Declaration of Helsinki were followed. Before starting the study, we obtained ethical approval from the Tehran University of Medical Sciences, and all subjects then signed an informed consent form (https://clinicaltrials.gov/ct2/show/NCT02833649).

### Patient enrolment criteria

During 6 months after CXL, the refraction was considered to be stable if there was a change in refraction of six subjective refractions within ±0.50 D of spherical equivalent.

#### Inclusion criteria

We considered the following as inclusion criteria: best corrected distance visual acuity (CDVA) of +0.4 logarithm of the minimum angle of resolution (log MAR) of 20/40 or better, K max ≤55, intraocular pressure (IOP) <20 mm Hg, clear cornea, normal ACD of at least 3 mm to the endothelium width of angle greater than 30°, a pupil diameter of less than 6.25 mm and a preoperative endothelial cell count related to age. Contact lens use was discontinued for at least 3 weeks for rigid lenses and 1 week for soft lenses before any intervention.

#### Exclusion criteria

Exclusion criteria included presentation of autoimmune diseases and other ophthalmic problems, except keratoconus, such as retinal degeneration, corneal scar or opacification, uveitis, cataract, glaucoma, diabetic retinopathy, central endothelial cell count of less than 2000 cells/mm^2^ by specular microscopy (SP-8800; Konan, Nishinomiya, Japan), central corneal thickness of less than 450 µm (measured by optical pachymetry (Pentacam-HR, Oculus Optikgerate, Wetzlar, Germany)) and ACD <3 mm from the endothelium to the anterior capsule measured by Orbscan IIZ (Orbscan, Bausch and Lomb, Rochester, New York, USA). The classification of keratoconus into four stages was based on the Amsler-Krumeich criteria^7^; in this practical classification, visual acuity, corneal power, thickness, transparency and astigmatism are considered. We calculate the size and axis of lens by STAAR nomogram (figure 1).

### Surgical procedure

#### CXL procedure

With the patient positioned under the operating microscope, an eyelid speculum was placed, and with a blunt spatula, the central 9 mm corneal epithelium was removed. The procedure has been illustrated based on Wollensak’s procedure; earlier on, tetracaine 1% and chloramphenicol 0.5% were instilled after informed consent was obtained. By using an epithelial spatula (Malosa Medical, Elland, UK), a 9 mm part of the central epithelium was removed. Five drops of riboflavin 0.1% in dextran 20% (Streuli Pharma, Uznach, Switzerland) were instilled and then reapplied after 5 min. After a period of 10 min, the eye was exposed to ultraviolet A (UVA). UVA exposure was performed for 30 min with 370 nm UVA radiation at 3 mW/cm^2^ with a beam diameter of 8 mm. During the procedure, riboflavin 0.1% drops were administered every 3 min; if the patient reported discomfort, 1% tetracaine drops were administered. Focus of the UVA beam over the axial cornea was monitored constantly. Neither intraoperative pachymetry nor slit-lamp examination was performed, because this was not part of the original treatment protocol.^8^ At the end of the procedure, the eye surface was washed with balanced salt solution, two drops of levofloxacin were instilled and a bandage soft contact lens was placed.

Patients used one drop of 0.3% ciprofloxacin four times per day for 7 days and 0.1% fluorometholone five times per day, with the dosage gradually tapering over 6 weeks postoperatively. After 4 days, the bandage soft contact lens was removed when epithelial healing was confirmed in slit-lamp examination. At 1 and 6 months postoperatively, complete evaluation was performed, and uncorrected distance visual acuity (UDVA), CDVA, refraction and anterior/posterior topography were included. The criteria for the progression of keratoconus were based on the following: (1) increase in the steepest K readings of at least 1.00 D in 1 year, as documented by corneal topography and/or in the Pentacam-HR (according to back surface progression indexes) and (2) deterioration of CDVA.^7^ Throughout the 6-month follow-up period, no sign of any further progression of keratoconus was recorded.

According to the results of the autorefractometer refraction and K readings, which are generally not precise in keratoconus and after CXL, all patients received a spectacle at least 6 months before ICL implantation. During follow-up after CXL, CDVA was obtained by focus curve for spherical component and rotating the astigmatism trial axis (Snellen chart). In spectacle administration, the maximum tolerable prescription of spherical equivalent was considered. ICL power was ascertained using the product given by the manufacturer, which was decided on the basis of the horizontal white to white (W–W) distance measured by Orbscan and with a calliper and VuMAX UBM (Sonomed, New York, USA). In addition, Sulcus to Sulcus (S–S) was measured by VuMAX UBM and Quantel Medical’s Linear 50 MHz UBM Probe. A minor clinical modification of ACD was performed by subtracting no more than 0.2 mm whenever corneal anterior bulging was advanced. Axial length measurement was recorded with LensStar^9^ (Haag-Streit, Koeniz, Switzerland).

### Implantable collamer lens insertion procedure

At least 12 months after CXL, the TICL implantation was performed. The pupil was dilated with cyclopentolate; to control cyclotorsion, the cornea was marked at four time periods (3, 6, 9 and 12 hours) by the surgeon (FD) through the slid-lamp examination in the upright surgical position. A 3.2 mm clear corneal temporal incision was made (regardless of the astigmatism axis) during the administration of stand-by anaesthesia. The anterior chamber was filled with sodium hyaluronate 1% hydroxypropylmethylcellulose. In the posterior chamber, the ICL was inserted through the incision using the injector cartridge given by the manufacturer, with consideration of the marks on the ICL (right superior and left inferior) to avoid the lens from being upside down. After alignment of the TICL and the proper intended axis in the sulcus, the remaining viscoelastic material was thoroughly removed from the anterior chamber with balanced salt solution. Eye drops containing 0.1% betamethasone and 0.3% ciprofloxacin eye drops were used four times per day for 10 days and then slowly tapered over 3 weeks.

### Study outcomes and patient follow-up

Postoperative examinations were conducted at a regular follow-up programme (baseline and at 1, 2, 4, 6 and 12 months and every 6 months thereafter to 4 years) between December 2011 and July 2015. The main outcomes parameters for this study were manifest and cycloplegic refractions, uncorrected distance visual acuity (UCDVA) and CDVA. We evaluated the following: anterior and posterior segments evaluation with dilated fundus examination, operative and postoperative complications, endothelial cell count measured on the central part of the cornea by specular microscopy (SP-8800, IOP with Goldman applanation tonometry and non-contact tonometer Topcon CT-1P. Vault height was measured subjectively (slit-lamp examination) and objectively with ultrasound *biomicroscope* (UBM).

### Evaluation of diagnostic *technologies*

Previous studies had concluded that aberrations are dynamic in nature. To evaluate tear film irregularity due to dry eye and fatigue and to analyse intersession repeatability, one experienced examiner (AS) measured the eyes five times successively. Measurements were rechecked by the same examiner from another set of eyes in two consecutive sessions 1 week apart to account for intersession reproducibility.^10^

Since we did not have access to the program Assort software (Assort) for vector analysis, we had to use the program Dr Peyman Calculator (http://www.drpeyman.ir/Ophthalmology_Calculator.htm), and graphical displays were performed using Microsoft Excel 2010 (Microsoft Corporation, Redmond, Washington, USA).

The tests for contrast sensitivity were performed under mesopic conditions for illumination (3 cd/m^2^) and photopic (85 cd/m^2^) using the MediWorks C901 Acuity Chart (Shanghai MediWorks Precision Instruments, Shanghai, China). The tests were performed with best spectacle correction before the operation and without correction after the operation, using a light level of 3 cd/m^2^ after 10 min of dark adaptation at a distance of 5.5 m. Testing was performed at 1, 3, 5, 6, 12 and 18 cycles per degree (c/d). The defocus curve was also obtained to evaluate the range of functional vision. Corneal, internal and ocular higher-order aberrations (HOAs) were measured. After evaluation, the best objective focus using the Optical Quality Analysis System (the HOA-derived modulation transfer function (MTF) and the root mean square of HOAs) was determined for a 6.0 mm pupil with the ray-tracing aberrometer (iTrace; Tracey Technologies, Houston, Texas, USA). Participants fixated on a near-infrared point light source during the measurements. The room illumination was 42 lx (digital lux metre, LX 1010 B). MTFs were measured for six spatial frequencies (5, 10, 15, 20, 25 and 30 C/D).

### Statistical analysis

To statistically analyse the results, we used the SPSS software (SPSS Statistics for Windows, V.23.0, 2013; IBM). The non-parametric Wilcoxon signed-rank test was applied to determine the significant differences between the objective results before and after the implantation of TICL, such as contrast sensitivity and the log MAR visual acuity defocus curve. Given that these factors had normal distribution, we report the mean and SD for them. Normal variables were reported as mean and SD, and we sat the median if distributions were skewed. We considered 5% level to find the statistically significant differences in our analysis.

## Results

### Patient population

**Figure 2:**
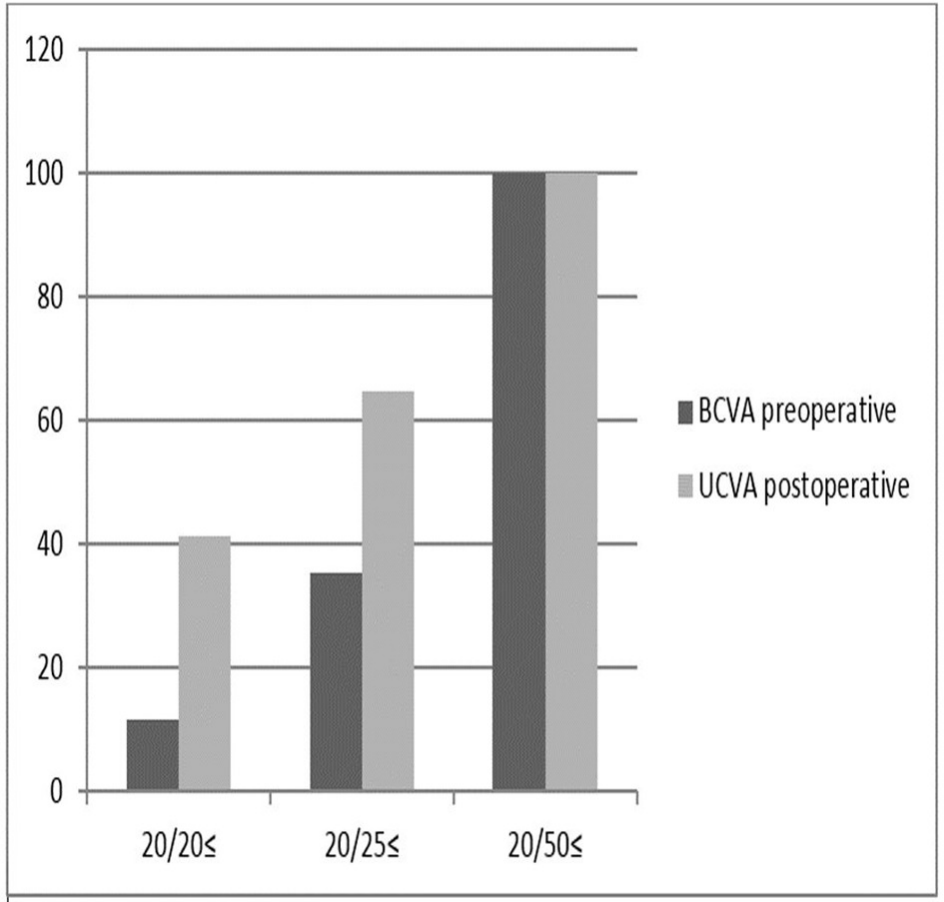
A comparison of preoperative best corrected distance visual acuity and postoperative uncorrected distance visual acuity 4 years after toric implantable collamer lens. UCVA, uncorrected visual acuity.

A summary of patient demographics is provided in table 1. The mean spherical error was −5.06±3.96 D (range: 0.00 to −18.00 D), and the cylindrical error was −3.57±1.56 D (range: −1.25 to −8.00 D). Patients at the time of surgery were aged 30.57±4.69 years (range 25–38 years). TICL was performed at the clinical investigational site from January 2011 to May 2012 in this group. Patients were followed up seven times after surgery at 1 month, 3 and 6 months and then 1, 2, 3 and 4 additional years. All patients had a preoperative uncorrected visual acuity (UCVA) worse than 20/40 with 95% having unaided acuity limited to counting of fingers. At 4 years, postoperative UCVA was better than or equal to preoperative CDVA in 92.50% (37/40) of eyes, and UCVA was increased by ≥1 lines in 25 eyes (table 2). The preoperative CDVA and postoperative UCVA at 4 years after TICL surgery were compared in figure 2. At 4 years postoperatively, 82.5% of eyes were within ±0.50 D, and 97.05% were within ±1.0 D of attempted correction, and the mean spherical and cylindrical manifest refractions were 0.44±0.40 D and −1.01±0.44 D, respectively. At the end of the follow-up, the mean vault was 658±54.33 µm (range, 500–711), and the IOP was 11.7±2.08 mm Hg.

**Table 1:** Preoperative and postoperative demographic data of patients undergoing toric implantable collamer lens surgery after 4 years

Parameter studied	Outcome
Refractive surgery (number of eyes)	24 patients; 40 eyes (16 patients bilateral, 8 patients unilateral)
Mean age±SD	30.57±4.69
Range	25–38
Gender	
Male	12 (50%)
Female	12 (50%)
Preoperative visual acuity (log MAR)	
UCVA	1.28±0.37 (range: 0.3 to 1.8)
CDVA	0.19±0.11 (range: −0.1 to 0.3)
Preoperative refractive error (spherical equivalent)	
Range	(−1.75 to −20.50)
Mean±SD	(−7.55±4.22)
Postoperative visual acuity (log MAR)	
UCVA	0.11±0.13
CDVA	0.04±0.16
Postoperative residual refraction (D)	
Mean±SD	0.44±0.40

CDVA, corrected distance visual acuity; log MAR, logarithm of the minimum angle of resolution; UCVA, uncorrected visual acuity.

**Table 2:** Manifest refraction, the toric implantable collamer lens

Preoperative				Postoperative after 4 years			
Cylinder	n/N (%)	Sphere	n/N (%)	Cylinder	n/N (%)	Sphere	n/N (%)
≤−1.50	4/40 (10%)	≤−3.00	12/40 (30%)	≤−1.50	36/40 (91.1%)	±0.25	15/40 (37.5%)
≤−3.50	20/40 (50%)	≤−5.00	20/40 (50%)	≤−3.50	40/40 (100%)	±0.50	24/40 (60%)
≤−5.00	34/40 (85%)	≤−10.00	34/40 (85%)	≤−5.00	40/40 (100%)	±0.75	37/40 (92.5%)
≤−7.00	37/40 (92.5%)	≤−12.00	37/40 (92.5%)	≤−7.00	40/40 (100%)	±1.00	39/40 (97.5%)
≤−8.00	40/40 (100%)	≤−18.00	40/40 (100%)	≤−8.00	40/40 (100%)	±1.25	40/40 (100%)
Mean±SD −3.57±1.56		Mean±SD −5.06±3.96		Mean±SD −1.01±0.34		Mean±SD 0.44±0.38	

### Safety

Log MAR CDVA was −0.11±0.11, −0.13±0.15, −0.13±0.15, −0.13±0.14 and −0.14±0.13 at 1 and 3 months and 1, 3 and 4 years after surgery with the ICL, respectively. We found a significant difference between preoperative CDVA ICL and all other follow-up (p<0.05, Wilcoxon signed-rank test). The safety index (mean postoperative CDVA/mean preoperative CDVA) was 0.45±0.56, 0.74±0.87, 0.71±0.92, 0.77±0.74 and 0.77±0.62 at 1 and 3 months and 1, 3 and 4 years after surgery with the ICL, respectively.

### Treatment effectiveness

UCVA at 4 years postoperatively for the entire population was 20/20 or better in 45% of eyes and 20/40 or better in 100% of eyes. The uncorrected visual results in this ‘all eyes’ group must be interpreted in the context: only 82.5% of these eyes had CDVA of 20/20 or better at the baseline. At 4 years, postoperative UCVA was equal to or better than preoperative CDVA in 95% (38/40) of eyes. Figure 2 compares the preoperative CDVA and postoperative UCVA at 4 years after TICL surgery.

### Predictability of manifest refraction (attempted vs achieved)

**Figure 3:**
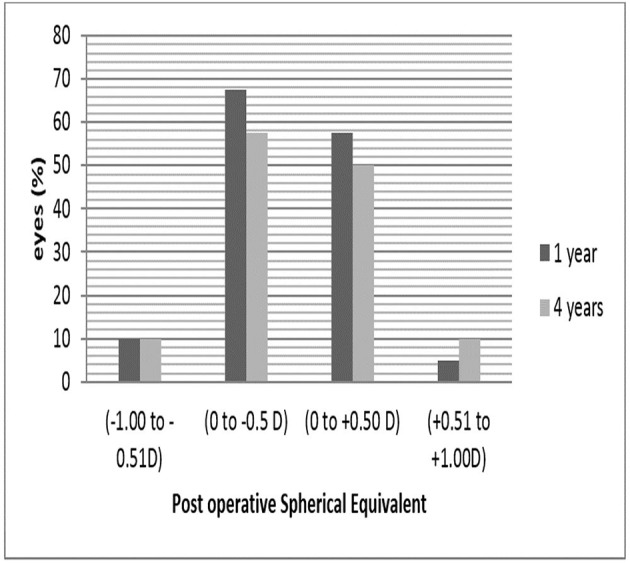
Postoperative spherical equivalent during follow-up (1 year and 4 years).

The following outcomes are expected to provide a more accurate assessment of refraction accuracy than the postoperative mean refractive spherical equivalent (MRSE) outcomes. At 4 years postoperatively, 82.5% of eyes were within ±0.50 D, and 97.05% were within ±1.0 D of attempted correction. The differences in SE, cylinder and sphere were statistically significant between preoperative and 1 month postoperatively. These differences remained stable 6 months and 1, 3 and 4 years after operation (figure 3). Even though emmetropia was the targeted postoperative refraction in all patients, small hyperopic and myopic deviations were found after TICL implantation.

### Stability

Spherical equivalent: 1 and 4 years after surgery, the mean manifest spherical equivalent was −0.07±0.5 and −0.01±0.48 D, respectively. The spherical equivalent significantly decreased from the baseline to 1 and 4 years (p<0.001, Wilcoxon signed-rank test). Changes in manifest refraction from preoperative to 1 year postoperative were −7.38±4.7 D and from preoperative to 4 years postoperative were −7.44±4.75 D. Astigmatism: 1 and 4 years after surgery, the mean manifest refractive cylinder was −1.03±0.58 and −1.01±0.48 D, respectively. Manifest astigmatism was significantly decreased from the baseline to 1 and 4 years (p<0.001, Wilcoxon signed-rank test). The change in manifest astigmatism from preoperative to 1 year postoperative was −2.77±1.71 D and from preoperative to 4 years postoperative was −2.79±1.78 D.

### Defocus curve

**Figure 4:**
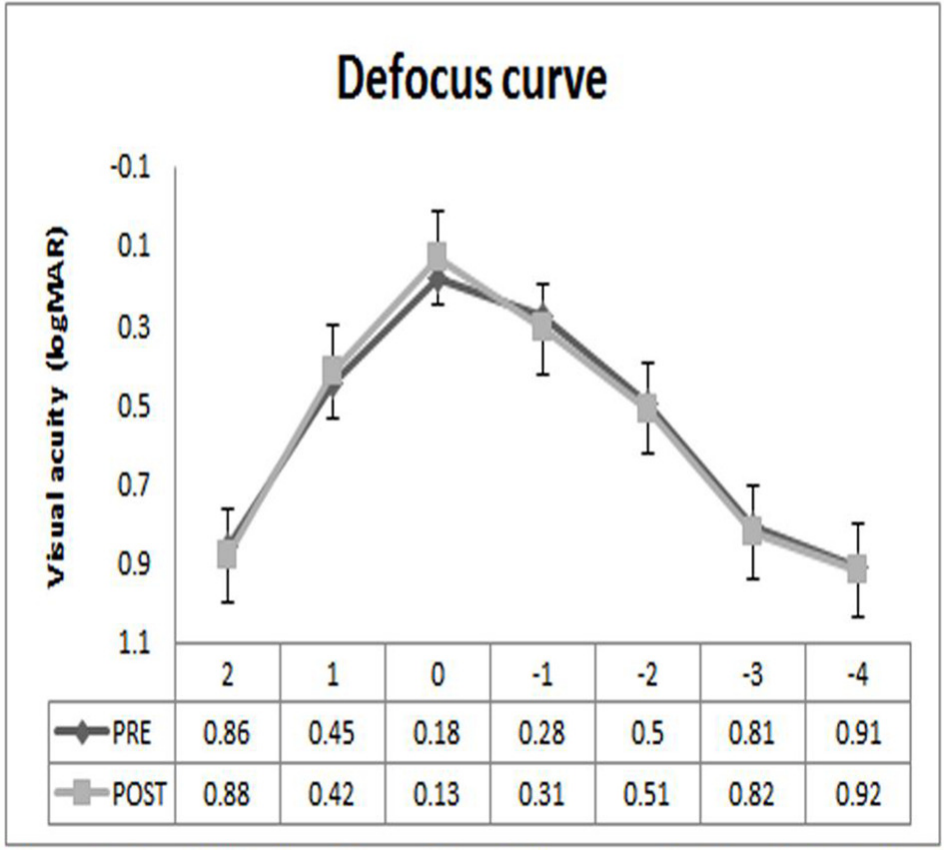
Log MAR (logarithm of the minimum angle of resolution) visual acuity defocus curve of +2, +1, 0, −1, −2, −3 and −4 D in a non-cycloplegic condition in the preoperative and postoperative.

Figure 4 shows the log MAR visual acuity under defocus curve of +2, +1, 0, −1, −2, −3 and −4 D in postoperative and preoperative periods in a non-cycloplegic condition. The differences between the measurements of binocular distance corrected defocus curve in the study demonstrated significant differences in log MAR visual acuity at the defocus curve levels of +1, 0 and −1 D, but no significant difference was observed at the defocus curve levels of +2,

−2, −3 and −4 D.

### Contrast sensitivity

**Figure 5:**
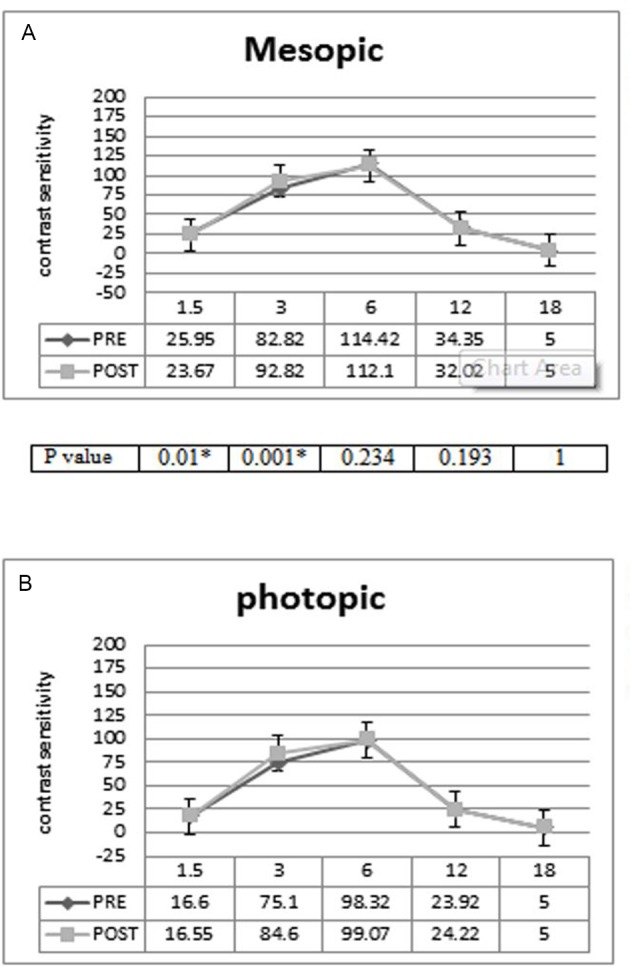
(A) Contrast sensitivity under mesopic illumination (3 cd/m^2^). *Statistically significantly different at a level of 0.05. (B) Contrast sensitivity under photopic illumination (85 cd/m^2^).

Figure 5A presents the mesopic contrast sensitivity results, which demonstrate no loss of contrast at any spatial frequency and a statistically significant improvement in contrast value at 3 and 1.5 per degree. In addition, photopic contrast sensitivity (figure 5B) demonstrated a significant improvement in contrast value at 3 per degree in similar mesopic conditions.

### Aberrometry

**Figure 6:**
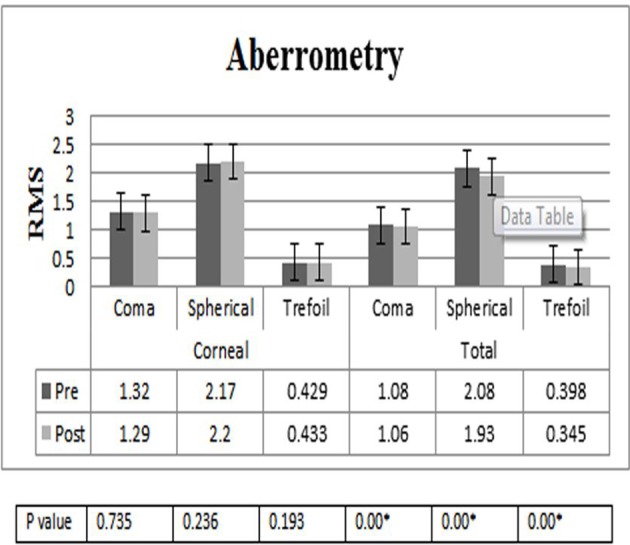
Total and corneal aberrations in 40 eyes with keratoconus before and after undergoing toric implantable collamer lens surgery. *Statistically significantly different at a level of 0.05. RMS, root mean square,

Preoperative and postoperative corneal and ocular aberrations for the current study, according to evaluation after 4 years, showed significant improvement in total aberrations after TICL implantation (figure 6).

### Astigmatism analysis by Alpin method

**Figure 7:**
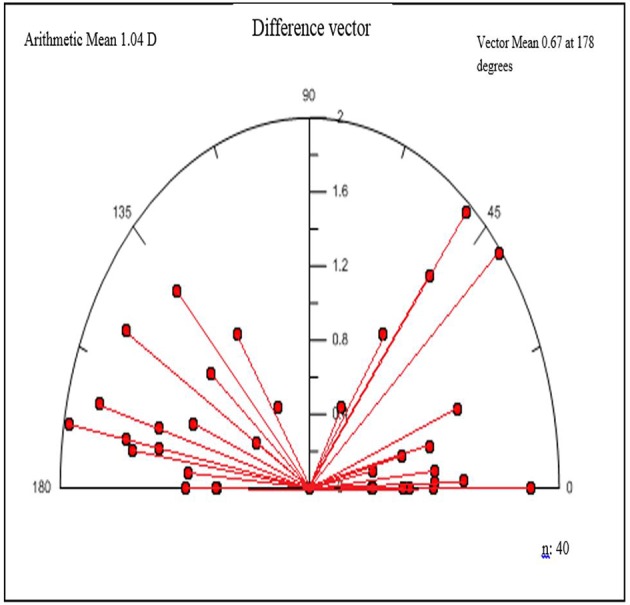
Vectorial display of the difference vector during postoperative follow-up.

Vector analysis of refractive astigmatism shows that the surgically induced astigmatism (SIA) (3.20±1.46 D, range 0.58 to 6.46 D) was not significantly different from the target induced astigmatism (TIA) (3.14±1.42 D, range 0.72 to 6.06 D) (p=0.620), but the mean difference vector (1.04±0.47 D, range 0.00 to 1.98 D) was different from zero (p=0.00). The mean magnitude of error was positive (overcorrection) and close to 0 (0.05±0.68 D, range −1.25 to 1.80 D), and the mean correction index was close to 1 (1.04±0.29 D, range 0.5 to 1.92 D) (figure 7).

### Modulation transfer functions

**Figure 8:**
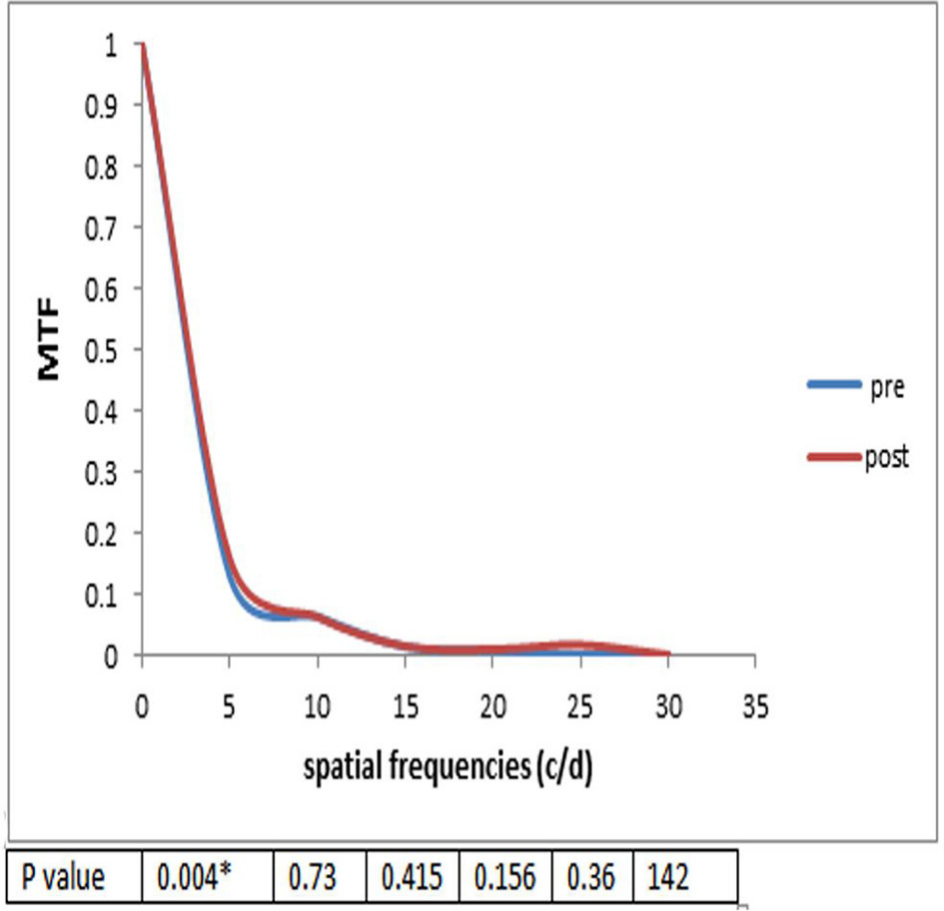
MTF pre-TICL and post-TICL implantation.

MTFs were estimated for six spatial frequencies (5, 10, 15, 20, 25 and 30 C/D) from the ray-tracing aberrometer at a pupil diameter of 6 mm. Figure 8 demonstrated a significant improvement in MTF value at 5 per degree (p=0.004).

### Intraocular pressure

The mean values of IOP changes were 11.67±2.3 mm Hg preoperatively and 13.45±1.75, 13.6±1.78, 12.37±1.59, 11.7±2.08 and 11.27±1.83 mm Hg at 1, 2 and 3 months and at 2 and 4 years, respectively. The mean values were determined using Goldman applanation tonometry and non-contact tonometer Topcon CT-1P at the postoperative follow-up periods. No significant difference was observed in the IOP change at the follow-up period.

### Endothelial cell substudy

The mean endothelial cell count (ECC) changed from 2426.58±107.64 cells/mm^2^ (range 2210 to 2577 cells/mm^2^) preoperatively to 2357.36±105.79 cells/mm^2^ (range 2146.6 to 2548 cells/mm^2^) after 4 years postoperative. At the end of follow-up, the mean ECC loss was ≤5%.

### Vaulting the TICL

Table 3 demonstrates the change in vault between consecutive measurements in different times. Subjective and objective vaults were stable after TICL implantation.

**Table 3:** Comparison of vault between each pair

	Value		Change		p Value
	Mean±SD	Range	Mean±SD	Range	
Vault.MONTH1	561.18±43.86	(450, 650)			
Vault.M2	644.08±45.45	(540, 712)	82.91±55.22	(−75, 230)	0.042
Vault.M6	672.49±41.79	(557, 725)	111.32±54.19	(−58, 240.3)	<0.001
Vault.Y1	658.45±98.22	(109, 720)	97.28±100.15	(−427, 239.3)	<0.001
Vault.Y4	641.02±136.31	(66, 721)	79.84±143.02	(−537, 240)	<0.001
Vault.M6_Vault.M2			28.41±18.54	(−2, 62.14)	<0.001
Vault.Y1_Vault.M2			14.37±101.69	(−603, 70)	<0.001
Vault.Y4_Vault.M2			−3.06±135.85	(−602, 71)	<0.001
Vault.Y1_Vault.M6			−14.04±95.23	(−601, 10)	>0.999
Vault.Y4_Vault.M6			−31.48±131.81	(−600, 12)	>0.999
Vault.Y4_Vault.Y1			−17.43±94.02	(−589, 2)	>0.999

All p values are based on Bonferroni-adjusted comparison between Vault MONTH1 with each other period.

Range: (minimum, maximum).

## Discussion

This study demonstrated the visual outcomes of TICL implantation in stable keratoconus with a long-term follow-up. Our observations were similar to those of other studies regarding the support safety, efficacy, predictability and stability of this procedure in patients with stable keratoconus (table 4).

**Table 4:** Peer reviewed publications on the use of TICL for refractive correction in corneal ectasia

Author(s) year, reference*PubMed, Scopus and science	Number of eyes, follow-up for each procedure	Preoperative data	Postoperative data	Complications	Final outcomes
Kamiya *et al,* J Refract Surg, 2008^44^	2 eyes (2 patients), 12 months	Case 1: −10.00–6.00×100 Case 2: −8.00–2.00×100	Case 1: +0.50–1.00×90 Case 2: −0.25–1.25×100	–	–
Alfonso *et al*, J Refract Surg, 2008^45^	25 eyes (16 patients), 12 months	Sph: −3.00 to −18.00 D Cyl: −0.50 to −3.00 D	UCDVA: 0.17±0.19 CDVA: 0.12±0.12	–	SI: 1.05 EI: 0.98
Alfonso *et al*, J Cataract Refract Surg, 2010^46^	30 eyes (21 patients), 12 months	Sph: −5.38±3.26 D Cyl: −3.48±1.24 D	Mean UDVA: 0.81±0.20 Mean CDVA: 0.83±0.18	–	SI: 1.16 EI: 1.07
Kamiya *et al*, Graefes Arch Clin Exp Ophthalmol, 2011^47^	27 eyes (14 patients), 6 months	SER: −10.11±2.46 D Cyl: −3.03±1.58 D	UCDVA: −0.09±0.16 CDVA: −0.15±0.09	–	SI: 1.12±0.18 EI: 1.01±0.25
Hashemian *et al*, Clin Exp Optom, 2013^5^	22 eyes (14 patients), 6 months	Sph: −4.98±2.63 D Cyl: −2.77±0.99 D	Sph: −0.33±0.51 D Cyl: −1.23±0.65 D UCVA: 0.76±0.23	–	SI: 1.40±0.32 EI: 1.24±0.34
Kymionis *et al*, Ophthalmic Surg Lasers Imaging, 2011^48^	1 patient, 3 months	UDVA: C.F CDVA: 0.7 log MAR	UCVA: 0.3 CDVA: 0.2	–	–
Fadlallah *et al*, J Refract Surg, 2013^9^ ^9^	16 eyes (10 patients), 6 months after CXL, 6 months after TICL	Mean UDVA: 1.67±0.49 CDVA: 0.15±0.06 log MAR SE: −7.24±3.53 D	Mean UDVA: 0.17±0.06 CDVA: 0.12±0.04 SE: −0.89±0.76 D	–	SI: 1.08±0.13 EI: 0.97±0.08
Coskunseven *et al*, Am J Ophthalmol, 2007^49^	3 eyes (2 patients). TICL minimum 6 months post-Intacs, 12 months	SE: −18.50±2.61 D	SE: 0.42 D	–	–
Fernandez-Vega L *et al*, Invest Ophthalmol Vis, 2010^50^	18 eyes (14 patients), 6 months	UDVA: 0.1±0.05 (decimal) CDVA: 0.7±0.19 (decimal)	UDVA: 0.6±0.19 CDVA: 0.8±0.14	–	–
Navas *et al*, Int Ophthalmol, 2012^51^	11 eyes (8 patients), 38.18±18.7 months	SE: −10.52±5.88 D UDVA: 1.31±0.37 log MAR CDVA: 0.28±0.14 log MAR	SE: −0.68±0.45 D UDVA: 0.14±0.04 CDVA: 0.16±0.08	–	SI: 1.28 EI: 0.88
Coskunseven *et al*, J Cataract Refract Surg, 2013^52^	14 eyes (9 patients), 12 months	UDVA: 0.01 (decimal) CDVA: 0.14 (decimal)SE: −16.40±3.56 D	UDVA: 0.44 CDVA: 0.57 SE: −0.80±1.02 D	–	–
Iovieno *et al*, Eur J Ophthalmol, 2013^53^	7 eyes (7 patients), 12.8±8.8 months	UCDVA: 1.18±0.4 SE: −8.09±3.77 D	UCDVA: 0.2±0.1 SE: −0.33±0.54	–	–
Kurian *et al*, J Cataract Refract Surg, 2012^54^	10 eyes (7 patients), 6 months	Mean SE: −7.21±2.25	Mean SE: −0.44±1.21 D	–	EI: 0.72 SI: 1.13
Ali M *et al*, Cornea, 2014^55^	29 eyes (16 patients), 3 months	CH: 9.2±1.4 mm Hg CRF: 8.4±1.6 mm Hg	CH: 8.8±1.3 mm Hg CRF: 8.8±1.6 mm Hg	–	–
Alió *et al*, J Cataract Refract Surg, 2014^6^	20 eyes received an iris claw phakic IOL, and 28 eyes a PC phakic IOL, 36 months	Mean SE: −9.31±4.20 (decimal) UDVA: 0.08±0.09 CDVA: 0.77±0.2	Mean SE: −0.46±0.88 (decimal) UDVA: 0.71±0.26 CDVA: 0.87±0.98	–	–
Antonios *et al*, J Ophthalmol, 2015^56^	30 eyes (19 patients), 2 years.	UDVA: 1.57±0.56 CDVA: 0.17±0.08	UDVA: 0.17±0.06 CDVA: 0.11±0.05	–	SI: 1.08±0.13 EI: 0.97±0.08
Kamiya *et al*, Br J Ophthalmol, 2015^2^	21 eyes (11 patients), 3 years	SE: −9.70±2.33 D UCVA: 1.46±0.15 log MAR	UDVA: −0.06±0.11 CDVA: −0.12±0.09	–	–
Dirani *et al*, Eur J Ophthalmol, 2014^57^	11 eyes (7 patients), 6 months	UCVA: 1.47±0.38 log MAR CDVA: 0.50±0.22 log MAR	UCVA: 0.27±0.20 CDVA: 0.19±0.11	–	–
Kummelil *et al,* SYMPOSIUM:KCN, 2013^58^	10 eyes (7 patients), 6 months	Mean SE: −7.21±2.25 D	Mean SE: −0.44±1.21 D	–	EI: 0.72 SI: 1.13
Park *et al*, Korean J Ophthalmol, 2013^59^	Case report, 37-year-old man, 20 months	Refraction: −12.0–3.5×30	Refraction: −1.75×180	–	–
Camoriano *et al*, J Cataract Refract Surg, 2012^60^	10 eyes (5 patients), 3 years	Mean SE: −6.71±0.9 D CDVA: 0±0.03 log MAR	SE: −0.58±0.1 D CDVA: −0.04±0.03	1 eye requiring removal and replacement of the toric phakic IOL	–
Emara *et al,* J Cataract Refract Surg, 2015^61^	11 eyes (9 myopic, 2 hyperopic), 16.8 months (in myopic eyes)	Mean SE: −11.07 D, SE: +8.75 D, CDVA: 20/171 for myopia CDVA: 20/130 for hyperopia	Mean SE: −1.40 D CDVA: 20/51	2 hyperopic eyes	–
Prakash *et al,* BMJ, 2015.^62^	Case report, 35-year-old man	Right UCVA: 4/60 CDVA: 6/18	Refraction: +0.50–0.50×85, UDVA: 6/12, CDVA: 6/9	–	–
Alfonso *et al,* J Cataract Refract Surg, 2011^63^	40 eyes (31 patients), 6 months	UDVA (decimal): 0.11±0.05 CDVA: 0.56±0.23 Sph: −7.56±7.27 Cyl: −4.19±2.08	UDVA (decimal): 0.50±0.27 CDVA: 0.73±0.20 SE: −1.19±1.33 D	–	EI: 0.88 SI: 1.28
Shaheen *et al,* Cornea, 2014^1^	16 eyes (n: 11), 3 years	UDVA: 0.63 CDVA: 0.56 (decimal)	UDVA: 0.88 CDVA: 0.89	–	–

CDVA, corrected distance visual acuity; CF, Count Fingers; CH, corneal hysteresis; CRF, corneal resistance factor; CXL, corneal collagen crosslinking; Cyl, cylinder; EI, efficacy index; log MAR, logarithm of the minimum angle of resolution; IOL, intra ocular lens; MCDVA, mean corrected distance visual acuity; MUDVA, mean uncorrected distance visual acuity; MSE, manifest spherical equivalent; PC, posterior chamber; SE, spherical equivalent; SER, spherical equivalent refraction; SI, safety index; Sph, sphere; TICL, toric implantable collamer lens; UCDVA, uncorrected distance visual acuity; UCVA, uncorrected visual acuity; UDVA, uncorrected distance visual acuity.

This technique is currently undergoing an approval procedure from the Food and Drug Administration in the USA. These findings in conjunction with excellent results for refractive indications that affect the quality of life^1^ enable TICL to be the first recommended phakic IOL approved in the USA for patient younger than 60 years.^11^

In our study, similar to the report of Gonzalez-Lopez, even amblyopic eyes (two cases) demonstrated significant improvement in UDVA and CDVA.^12^

The visual acuity test is gradually becoming the gold standard for the assessment of vision, providing solely a restricted quantity of data under artificial conditions. Contrast sensitivity testing presented a variety of visual performance data under genuine conditions.^13 14^ This motivated us to make progress in this area of medicine. To the best of our knowledge (PubMed and Web of Science), this is the first study of TICL in patients with stable keratoconus (not limited to mild and moderate) with a long follow-up that focuses on visual quality indexes (contrast sensitivity, MTF, defocus curve and aberrations) in January 2011.

The amount of aberrations in the eye is related to factors such as age, refraction, severity of keratoconus and even techniques of evaluation.^15–22^ In the current study, after 4 years of follow-up evaluation of aberration by ray-tracing technology, there was a significant improvement in total aberrations after TICL implantation in comparison with previous surgery, and also MTFs were appraised for six spatial frequencies (5, 10, 15, 20, 25 and 30 c/d) from the ray-tracing aberrometer at a pupil diameter of 6 mm improvement in MTF value at 5 per degree (*p=0.004). An asymmetrically blurred retinal image exerted by the higher-order aberrations in keratoconic eyes with TICL seems to compensate through mechanisms such as the neural visual system and other related components that aid in improving long-term visual experience.^23–26^

No loss in contrast sensitivity was seen at any spatial frequency. Mesopic contrast showed a statistically significant improvement in value at 3 and 1.5 per degree, and the photopic contrast sensitivity showed a significant improvement in contrast value at 3 per degree similar to mesopic conditions.

There is no completely perfect test for contrast sensitivity.^14^ We chose this method, because it is user-friendly, time-saving and available, and it reduces the examiners’ error. However, despite all the considerations, the test outcome was influenced by many other factors that improved contrast sensitivity, besides refractive correction of secondary procedure, one-time CXL with riboflavin and UVA, improve contrast sensitivity and aberrations.^27 28^ We had three patients with preoperative astigmatism greater than six (table 2). A few patients had better tolerance to myopic defocus curve (−1), which seems related to the residual refraction in these patients. Owing to the Alpin analysis of the astigmatism changes, we used the vector technique to assess the magnitude and axis refractive astigmatism variety with the surgery. The mean angle of error indicated that the mean angle of the SIA vector was 0.19±9.88° counter-clockwise to the TIA vector. If the treatment is 100% effective, this vector would be 0. The torque measure of astigmatic change was induced by SIA owing to misalignments of the surgery. In our study, a torque vector of 0.2 D was acquired. The result is close to 0 and shows a trace of astigmatic change induced by SIA.

The major probable complications after ICL implantation are cataract formation,^29^ acute increase in IOP and night vision disturbance.^30^ Anterior segment anatomy evaluation with new advanced technologies and attention of the surgeon to warning signs before surgery allow the selection of the proper size and decrease probable complications.^31^ We think that before surgery, older patients and patients with shallower ACD and larger WTW (White to White) should be made aware of the probability of complication after this procedure^32^. However,cataract surgery in keratoconus leads to revitalisation of visual acuity, especially by different means such as toric and toric multifocal lenses.^33^ Possible risk factors for night vision disturbances after ICL are WTW diameter of the cornea, difference between the optic zone diameter and the mesopic pupil size, halo and toricity of the ICL and glare.^34^ The preoperative and postoperative screening consisted of a complete ophthalmic examination (figure 1).

The ICL implantation is as a feasible approach with less encroachment in visual performance, because it does not change the curvature ratios between the anterior and posterior corneas.^35^ In this respect, although some approaches may show slightly better outcomes for UCVA and refractive predictability, in a study by Alfonso, TICL implantation showed reliable results similar to those of bioptics. A single procedure with TICL implantation may avoid the potential complications for alternative second surgical procedures.^36^

A trend toward decrement of corneal transplantation for keratoconus comparing two different periods was reported by some studies.^37 38^ It is a promise that seems related to contemporary management modalities in earlier detection of progression^7^ and treatments of keratoconus. Divorce commercially available riboflavin (more potent riboflavin with fewer cytotoxicity), better protocols and techniques of crosslinking ‘the procedure in halting the progression of the disease’^39–42^ can enable the correction of visual defects in patients with keratoconus, thus TICL implantation becomes a perfect refractive surgical correction technique in the future.^43^
